# Eight weeks of high-intensity interval vs. sprint interval training effects on overweight and obese adolescents carried out during the cool-down period of physical education classes: randomized controlled trial

**DOI:** 10.3389/fpubh.2024.1394328

**Published:** 2024-04-30

**Authors:** Noelia González-Gálvez, Antonio Soler-Marín, Tomás Abelleira-Lamela, Lucia Abenza-Cano, Adrián Mateo-Orcajada, Raquel Vaquero-Cristóbal

**Affiliations:** ^1^Facultad del Deporte, UCAM Universidad Católica de Murcia, Murcia, Spain; ^2^Facultad de Farmacia y Nutrición, UCAM Universidad Católica de Murcia, Murcia, Spain; ^3^Department of Physical Activity and Sport, Faculty of Sport Sciences, University of Murcia, San Javier, Spain

**Keywords:** physical activity, exercise, blood pressure, HIIT, SIT, puberty, metabolic diseases, physical education

## Abstract

**Objective:**

The aim of this study was to evaluate the effect of sprint interval training (SIT) and [high intensive interval training (HIIT)] carried out during the cool-down period of the physical education classes on body composition, blood pressure variables (BP) and pulse rate (PR), and cardiorespiratory fitness of adolescents who are overweight and obese, and to compare the differences in enjoyment in response to SIT vs. HIIT.

**Methods:**

For this randomized controlled trial, forty-five adolescents were recruited from a high school and were randomly placed into three groups. SIT and HIIT trained for 8 weeks, twice a week, for 12 min/session. Experimental group (EG) 3 was the control, and they maintained their regular physical education class schedule. The SIT group performed 6 sets of 60 s of work (90-95%HRmax) / 60 s of rest (50-55%HRmax), and the HIIT group performed 3 sets of 2 min of work (80-85%HRmax) / 2 min of rest (50-55%HRmax).

**Results:**

Both experimental groups showed a significant improvement in fat mass (FM) (%) and trunk FM (kg). In addition, EG2 reported a significance improvement in lean mass (kg), blood pressure BP (mmHG), systolic blood pressure (SBP) (mmHg), diastolic blood pressure (DBP) (mmHg), PR (bpm), and VO^2^max (ml/kg/min).

**Conclusion:**

The present study found that a HIIT protocol performed during the cool-down period of the physical education classes generated adaptations such as improvement in body composition, BP variables and PR, and cardiorespiratory fitness, in overweight and obese adolescents. In contrast, the group of overweight and obese adolescents who performed SIT showed limited benefits, with changes in fat mass only.

## 1 Introduction

Obesity in children and adolescents is a global health issue with increasing prevalence in the last decades (1), which is particularly worrying considering that childhood and adolescence are the stages in which lifestyle habits are set (2). Numerous studies have shown that levels of physical activity decrease during childhood and adolescence (3), reaching high rates of physically inactive children and adolescents according to the criteria of the World Health Organisation (WHO) (4–6). In addition, the percentage of physically inactive individuals is even higher among overweight and obese children and adolescents (7).

High intensity interval training (HIIT) has been presented as an alternative with which to increase the practice of physical activity at high intensities, as this type of training minimizes the time spent in the training session (8). More specifically, HIIT-based physical exercise programmes have been presented as a good option to improve the level of cardiorespiratory fitness (CRF) of overweight and obese adolescents, showing significantly improves maximal oxygen uptake (mean change: 0.51 to 1.117 mL.kg^-1^.min^−1^; *p* < 0.001) (9–11).

More discrepancies exist about its effectiveness body composition and cardiometabolic risk factors, as there is a great heterogeneity and inconsistency in the results regarding these variables (9, 11, 12), which could be due to differences in the training intensity of the exercise performed (9, 12). More specifically, a systematic review with meta-analysis showed a significant decrease in body mass (mean change: −0.295 kg; *p* = 0.012) and fat mass (mean change: −0.786 kg; *p* = 0.021) after a HIIT programme in overweight and obese children and adolescents; but not in body mass index (BMI) (*p* = 0.061), nor in fat-free mass (*p* = 0.625) (9). It also found a significant decrease in systolic (SBP) and diastolic blood pressure (DBP) (mean change: −1.026 and-0.966 mmHg; *p* < 0.001) (9). Another systematic review with meta-analysis looked at the effect of HIIT on adult population with different weight statuses (12). It was found that there was a significant decrease in fat mass (mean change: −0.189 kg; *p* = 0.003), abdominal fat mass (mean change: −0.188 kg; *p* = 0.007) and visceral fat mass (mean change: −0.243 kg; *p* = 0.018) after the HIIT program (12).

Indeed, a systematic review with meta-analysis that analysed the effects of HIIT on total, abdominal, and visceral fat mass in normal-weight and overweight/obese adults found that the adaptations of this type of training may depend on the intensity at which the exercises are performed (12). Another systematic review on HIIT in children and adolescents found that the effectiveness of this type of programme on physiological parameters, such as blood pressure (BP) and pulse rate (PR), may also depend on the intensity at which the exercises is performed, the duration of the exercises performed and the rest intervals (13). For this reason, different protocols that apply higher intensities and shorter durations have been investigated. These are called Sprint Interval Training (SIT) (14, 15) protocols.

In this sense, a maximum or supramaximal intensity with short series should affect the neuromuscular system, while submaximal intensities close to maximum will mainly improve cardiorespiratory and cardiovascular fitness (14). Regarding the effects on cardiorespiratory fitness, a systematic review reported that HIIT and SIT protocols generate similar adaptations in adults (16), although the effects on other variables are different (17), and their effects on adolescents have not been investigated. On the other hand, these systematics reviews with meta-analysis have some limitations, such as the lack of quality of the included studies, and few studies followed a randomized controlled trial design (18). However, there is only one study that compares de effect of SIT vs. HIIT protocols in obese preadolescent boys on vascular function (19).

Research in this regard in the school environment applies different protocols, the most used being the protocols between 60 and 120 s (10, 11, 20). Protocols with durations of 30 s have been widely questioned given their low viability by showing worse adherence rates associated with negative feelings (21) among children and adolescents. On the other hand, to achieve the appropriate intensities (22), it is necessary to use specialized equipment. On the other hand, high values of enjoyment and satisfaction in adolescents have been demonstrated in protocols that apply series durations of 1 min (23). Some recent research has focused on submaximal protocols of one to 2 min of duration (10, 20). In the school environment, there are few investigations that apply 4-min periods, because this requires longer session durations, making it not feasible to be applied in the school environment. In this sense, it is necessary to compare protocols that may be susceptible to use in the school environment.

Thus, the aim of this study was to evaluate the effect of SIT and HIIT carried out during the cool-down period of the physical education classes during school hours on body composition, BP variables and PR, and cardiorespiratory fitness of adolescents who are overweight and obese, and to compare the differences in enjoyment in response to SIT vs. HIIT. Based on the findings of previous studies, it was hypothesised that both SIT and HIIT would have positive effects on body composition, BP, PR, and cardiorespiratory fitness in adolescents with overweight and obese, compared to the control group. However, the absence of previous studies comparing SIT vs. HIIT protocols in these overweight and obese adolescents leads to hypothesise that there will be similar adaptation in terms of cardiorespiratory fitness, without a clear hypothesis on what will happen to body composition and BP variables or PR.

## 2 Materials and methods

### 2.1 Study design

The trial design was registered with ClinicalTrial.gov (Code: NCT05544370) and followed the Consolidated Standards of Reporting Trials (CONSORT) guidelines and the Template for Intervention Description and Replication (TIDIER) checklist. All the adolescents and parents/tutors signed an informed consent form. This project was developed with a research grant from the Universidad Católica de Murcia Research Projects Program (PMAFI-11/19) and through a contract/agreement with the Archena City Council (CO/AY/58–20). Ethical approval for this study was obtained from the Universidad Católica de Murcia (CE061914; 09/05/2019) and was implemented according to the guidelines for human research of the Helsinki Declaration.

### 2.2 Participants

The study participants were recruited form a high school in Murcia (Spain). The inclusion criteria were (a) being in their initial years of Compulsory Secondary Education; (b) being physically active in physical education sessions; (c) having a body mass index (BMI) to be considered overweight or obese according to Cole et al. (24); (d) agree to participate in the study and sign the parent/guardian and adolescents consent form; (e) be present at the time of the assessments; and (f) no changes in out-of-school physical activity during the intervention period. The exclusion criteria were (a) presenting any musculoskeletal, neurological, cardiological, metabolic, or rheumatic pathologies prior to the intervention or at any time during the intervention, and (b) changing schools.

A total of 45 adolescents (12.51 ± 0.75 years old), who met the inclusion criteria, were recruited and randomly placed into three groups. A stratified randomization method (Microsoft Excel 2016) was used for the subject’s distribution in SIT (*n* = 15), HIIT (*n* = 15) and control group (CG) (*n* = 15) according to CRF. Once the pre-test was implemented, the participants were ordered from lowest to highest cardiorespiratory fitness and three quartiles were made according to their levels of cardiorespiratory fitness. Each participant was categorized according to the level of cardiorespiratory fitness obtained and then a randomized sequence was generated for these three groups. The group assignment was blinded to the examiner and the staff who performed the statistical analysis.

**Figure 1:**
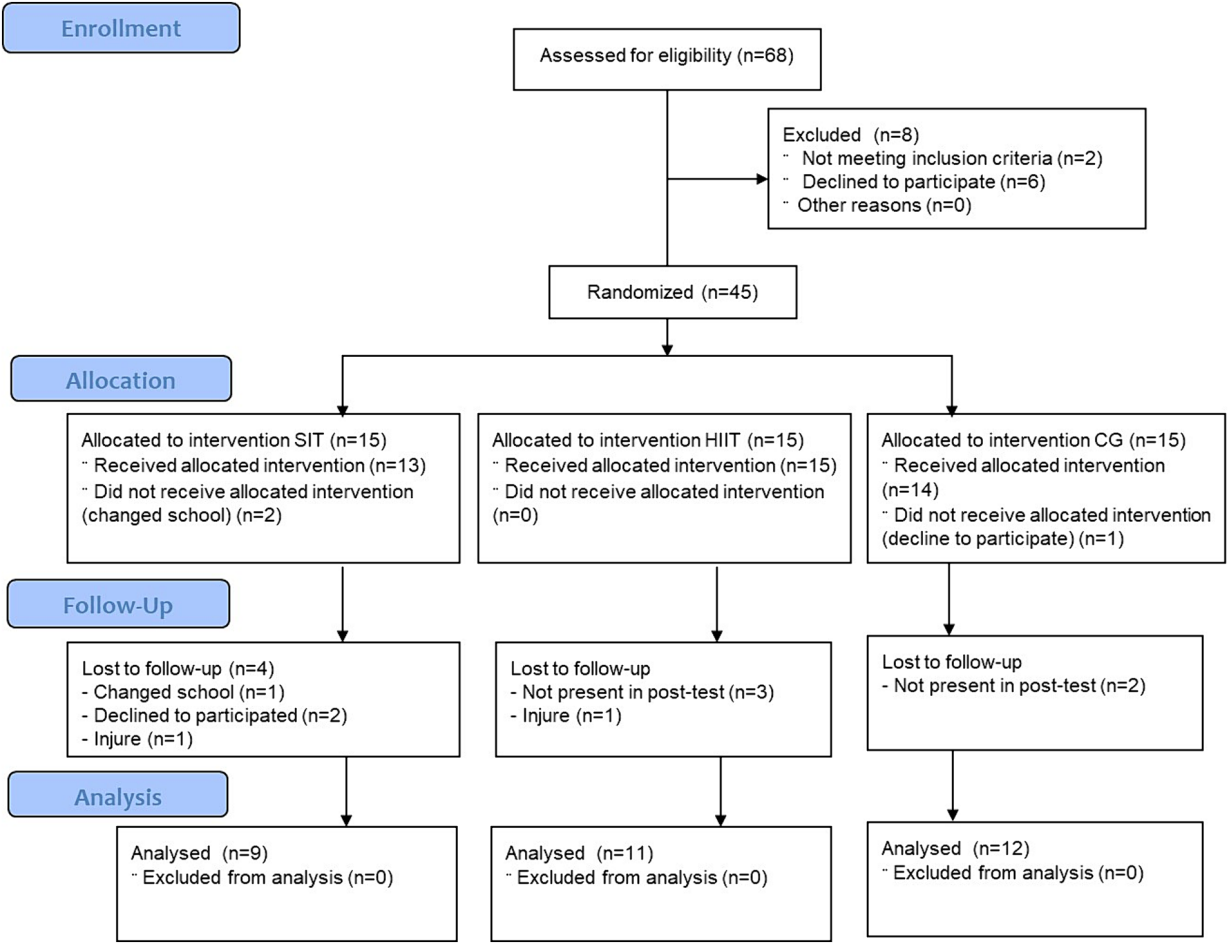
Consolidated standards of reporting trials flow diagram. SIT, experimental group 1; HIIT, experimental group 2; CG, control group.

The calculations to establish the sample size were performed using Rstudio 3.15.0 software (Posit, PBC, Boston, Massachusetts). The significance level was set at α = 0.05 and the power to 95% (1-β = 0.95). The standard deviation was used, according to the standard deviation for cardiorespiratory fitness in previous studies (25). To detect the minimum clinically-significant change of a total of 4.064 mL/kg/min (26), a total of 30 participants were required. Considering a dropout rate of up to 6%, at least 32 participants were needed. The final study sample consisted of 32 adolescents. This sample provided a power of 95% if values were found between and within a variance of 3.97 mL/kg/min in the test. Figure 1 shows the CONSORT flow diagram. The final sample consists of 9, 11 and 12 participants for SIT, HIIT and CG, respectively (SIT = male: 6, female: 3; HIIT = male: 6, female: 5; CG = male: 6, female: 6) (14).

### 2.3 Assessment

#### 2.3.1 Conditions

The same trained researchers measured the variables. The participants were measured in a single session between 08:00 and 11:00 am. The laboratory temperature was standardised at 24°C. The children were instructed to wear lightweight clothes. No warm-up or stretching were performed before the measurements, and there was a 5-min rest between tests.

#### 2.3.2 Anthropometric and body composition variables

Body mass (TANITA BF-522 W, Tokyo, Japan), and stretch stature (SECA 217 stadiometer, Germany) were measured, and BMI was calculated using these data (24). Total fat mass (FM) (percentage), trunk FM (kg), and lean mass (kg) were measured with a portable digital scale (TANITA BF-522 W, Tokyo, Japan).

#### 2.3.3 Blood variables

SBP, DBP, PR were measured with an automatic device (OMRON, model HEM-7113). Measurements were taken on the left arm twice at 5-min intervals, with the participant in a sitting position. Mean BP was calculated with the following formula: 1/3(SBP − DBP) + DBP. BP is used in clinical practice, evaluation protocols, and in a previous study as a mediation variable; it allows blood pressure to be used as a single variable (27).

#### 2.3.4 Cardiorespiratory fitness

The Course-Navette test (a 20-m Shuttle run test) was used to assess CRF. The protocol published in previous publications was followed (28). The maximum oxygen consumption (VO2max, ml/kg/min) was estimated from the number of laps performed by the test participants using the equation reported by Leger et al. (28) (VO^2^max = 31.025 + 3.238X − 3.248A + 0.1536AX where X = running speed and A = age).

#### 2.3.5 Physical activity enjoyment

Enjoyment was assessed using the Physical Activity Enjoyment Scale (PACES) questionnaire. This questionnaire consisted of 16 items, which were answered with a 5-point Likert scale, ranging from 1 (strongly disagree) to 5 (strongly agree). The Cronbach’s alpha value of the original scale was 0.89 (29). The Cronbach’s alpha value was 0.76 in the present study, indicating a good internal consistency.

### 2.4 Intervention

In order to develop the protocols, the classification suggested by Bucchet and Laursen (14) was utilized, which considered SIT as an effort of short duration (≤60 s) with a maximum intensity, and HIIT as a longer effort (>60 s up to 5 min) and an intensity close to the maximum. The SIT and HIIT groups trained twice a week for 8 weeks, at school during the Physical Education sessions, after a conventional warm-up. The protocol for SIT and HIIT is presented in Table 1. The work consisted of running. Heart rate (HR) was monitored by means of the Polar TM M430 HR monitor, and participants were encouraged to achieve the prescribed heart rate in each protocol. The CG students attended their regular physical education sessions.

**Table 1:** Exercise training data for the SIT and HIIT groups.

	Weeks 1–4	Weeks 5–8
**SIT (SIT)**		
Work-rest interval duration (s)	60:60	60:60
Work-Rest interval intensity (RHR)	90–50%	95–55%
Number of sets	6	6
Duration (min)	12	12
**HIIT (HIIT)**		
Work-rest interval duration (s)	120:120	120:120
Work-rest interval intensity (RHR)	80–50%	85–55%
Number of sets	3	3
Duration (min)	12	12

SIT, sprint intensity training; HIIT, high intensity interval training; SIT, experimental group1; HIIT, experimental group 2; s, seconds; min, minutes; RHR, reserve heart rate.

### 2.5 Statistical analysis

The Kolmogorov–Smirnov test and Mauchly’s W-test were used to assess the normality and the sphericity of the data.

The primary analysis performed was an analysis of covariance, which was used to compare the changes from the baseline between groups. This analysis was performed unadjusted and adjusted by sex as in previous research studies (30, 31). A two-way ANOVA with repeated measures in 1 factor x 2 factors (time * group) was used to analyse inter-and intra-group differences. The effect size was calculated using partial eta-squared (η2p) for analysis of variance, and will be defined as small: ES ≥ 0.10; moderate: ES ≥ 0.30, large: ES ≥ 1.2; or very large: ES ≥ 2.0, and an error of *p* ≤ 0.05 is established (32). All analyses were based on intention-to-treat, with an error of *p* ≤ 0.05 established. The statistical analysis was performed using the statistical package SPSS 24.0 for Windows (IBM, Armonk, NY). Generalisability allows measuring the reliability of a test by quantifying the importance of each of its sources of variability. In a complementary way, a generalisability analysis was carried out to assume that the estimated results were reliable and generalisable with the SAGT v1.0 software (MenPas, Madríd, España) (33).

## 3 Results

Table 2 shows the unadjusted results and Table 3 show the result adjusted by sex. After adjusting the analysis for sex, SIT and HIIT showed a significant improvement in FM (%) and trunk FM (kg). In addition, HIIT reported a significance improvement in lean mass (kg), BP (mmHg), SBP (mmHg), DBP (mmHg), PR (bpm), and VO^2^max (ml/kg/min).

**Table 2:** Differences between groups in the change pre-post intervention and interaction group*time.

Outcome	Group	Pre-test (M ± ED)	Post-test (M ± ED)	Non-adjusted by sex			Group * time interaction		
				Difference post-pre (M ± ED)	*p*	95% CI (Mpost-Mpre)	*F*	Sig	ES
BMI (kg/m^2^)	SIT	25.1 ± 1.1	24.7 ± 1.0	−0.4 ± 0.3	0.158	−1.046; 0.179	0.259	0.774	0.020
	HIIT	22.9 ± 1.0	22.7 ± 1.0	−0.2 ± 0.3	0.529	−0.761; 0.401			
	CG	26.3 ± 1.1	25.9 ± 1.	−0.4 ± 0.3	0.158	−1.046; 0.179			
FM (%)	SIT	29.2 ± 2.3	26.7 ± 2.6	−2.5 ± 0.7	0.003	−3.969; −0.942	1.437	0.257	0.103
	HIIT	26.5 ± 2.2	24.3 ± 2.4	−2.2 ± 0.7	0.004	−3.656; −0.784			
	CG	31.9 ± 2.3	31.0 ± 2.6	−0.8 ± 0.7	0.268	−2.347; 0.680			
Lean mass (kg)	SIT	46.0 ± 2.8	46.7 ± 2.7	0.7 ± 0.5	0.152	−0.283; 1.728	2.190	0.133	0.149
	HIIT	40.2 ± 2.7	42.0 ± 2.6	1.8 ± 0.5	0.001	0.886; 2.794			
	CG	42.4 ± 2.8	43.0 ± 2.7	0.6 ± 0.5	0.266	−0.450; 1.561			
Trunk FM (kg)	SIT	5.3 ± 1.0	4.5 ± 0.9	−0.8 ± 0.2	0.000	−1.250; −0.416	2.732	0.085	0.179
	HIIT	4.0 ± 0.9	3.4 ± 0.9	−0.6 ± 0.2	0.008	−0.946; −0.154			
	CG	5.1 ± 1.0	4.9 ± 0.9	−0.2 ± 0.2	0.418	−0.584; 0.250			
BP (mmHg)	SIT	80.9 ± 5.2	81.9 ± 2.3	1.0 ± 5.7	0.857	−10.767; 12.851	1.878	0.176	0.140
	HIIT	95.1 ± 4.7	82.0 ± 2.1	−13.1 ± 5.1	0.018	−23.629; −2.504			
	CG	89.8 ± 5.2	87.1 ± 2.3	−2.7 ± 5.7	0.645	−14.476;9.142			
SBP (mmHg)	SIT	119.9 ± 6.8	123.0 ± 3.8	3.1 ± 6.9	0.654	−11.091; 17.341	1.396	0.268	0.108
	HIIT	128.1 ± 6.1	116.3 ± 3.4	−11.8 ± 6.1	0.067	−24.516;0.916			
	CG	128.3 ± 6.8	126.5 ± 3.8	−1.8 ± 6.9	0.801	−15.966; 12.466			
DBP (mmHg)	SIT	61.4 ± 5.5	61.4 ± 2.6	0.0 ± 6.1	1.000	−12.705; 12.705	1.567	0.230	0.120
	HIIT	78.6 ± 4.9	64.9 ± 2.3	−13.7 ± 5.5	0.020	−25.064; −2.336			
	CG	70.5 ± 5.5	67.4 ± 2.6	−3.1 ± 6.1	0.616	−15.830;9.580			
PR (bpm)	SIT	79.1 ± 4.6	74.5 ± 4.5	−4.6 ± 3.3	0.180	−11.542; 2.292	2.381	0.115	0.172
	HIIT	84.7 ± 4.1	74.2 ± 4.0	−10.5 ± 3.0	0.002	−16.687; −4.313			
	CG	81.3 ± 4.6	80.4 ± 4.5	−0.9 ± 3.3	0.796	−7.792; 6.042			
Chol (mg/dl)	SIT	148.4 ± 15.7	146.8 ± 14.0	−1.6 ± 3.9	0.689	−10.342;7.142	0.012	0.916	0.001
	HIIT	170.0 ± 14.4	167.8 ± 12.8	−2.2 ± 3.5	0.554	−10.147; 5.814			
LDL (mg/dl)	SIT	56.0 ± 10.3	60.0 ± 9.1	4.0 ± 5.4	0.480	−8.274; 16.274	5.892	0.038	0.396
	HIIT	83.2 ± 9.4	69.3 ± 8.3	−13.8 ± 5.0	0.021	−25.038; −2.628			
HDL (mg/dL)	SIT	60.2 ± 8.8	59.4 ± 8.4	−0.8 ± 2.5	0.758	−6.491; 4.891	3.985	0.077	0.307
	HIIT	68.2 ± 8.1	74.2 ± 7.7	6.0 ± 2.3	0.028	0.805; 11.195			
Trig (mg/dl)	SIT	164.0 ± 17.9	136.4 ± 29.9	−27.6 ± 32.7	0.420	−101.487; 46.287	1.667	0.229	0.156
	HIIT	93.0 ± 16.3	122.5 ± 27.3	29.5 ± 29.8	0.348	−37.949; 96.949			
LipidNonHDL (mg/dl)	SIT	88.8 ± 11.7	87.4 ± 12.5	−1.4 ± 3.3	0.684	−8.941; 6.141	2.248	0.168	0.200
	HIIT	101.8 ± 10.7	93.7 ± 11.4	−8.2 ± 3.0	0.025	−15.050; −1.283			
Chol/HDL (mg/dl)	SIT	2.5 ± 0.2	2.6 ± 0.3	0.0 ± 0.1	0.882	−0.277; 0.317	2.909	0.122	0.244
	HIIT	2.6 ± 0.2	2.4 ± 0.2	−0.3 ± 0.1	0.042	−0.555; −0.012			
VO^2^max (ml/kg/min)	SIT	23.2 ± 1.0	24.2 ± 1.2	1.0 ± 0.6	0.116	−0.272; 2.296	3.422	0.051	0.237
	HIIT	22.8 ± 1.0	25.0 ± 1.2	2.2 ± 0.6	0.002	0.942; 3.510			
	CG	20.6 ± 1.0	20.6 ± 1.1	0.0 ± 0.6	1.000	−1.211; 1.211			

BMI, body mass index; BP, blood pressure; Chol, cholesterol; DBP, diastolic blood pressure; FM, fat mass; HDL, high density lipoprotein; LDL, low density lipoprotein; PR, pulse rate; SBP, systolic blood pressure; Trig, triglycerides.#Tendency towards significance from corresponding CG value at the *p* < 0.07 level. ^*^Significantly different from corresponding CG value at the *p* < 0.05 level. ** Significantly different from corresponding CG value at the *p* < 0.01 level.

**Table 3:** Differences between groups in the change pre-post intervention and interaction group*time adjusted by sex.

Outcome	Group	Adjusted by sex			Group * time interaction		
		Difference post-pre (M ± ED)	*p*	95% CI (Mpost-Mpre)	*F*	Sig	ES
BMI (kg/m^2^)	SIT	−0.5 ± 0.3	0.120	−1.116; 0.137	0.292	0.750	0.024
	HIIT	−0.2 ± 0.3	0.530	−0.763; 0.403			
	CG	−0.4 ± 0.3	0.226	−1.004; 0.250			
FM (%)	SIT	−2.2 ± 0.7	0.005	−3.712; −0.729	0.848	0.441	0.066
	HIIT	−2.2 ± 0.7	0.003	−3.608; −0.832			
	CG	−1.1 ± 0.7	0.152	−2.560; 0.423			
Lean mass (kg)	SIT	0.6 ± 0.5	0.228	−0.408; 1.632	2.194	0.133	0.155
	HIIT	1.8 ± 0.5	0.001	0.890; 2.790			
	CG	0.7 ± 1.0	0.190	−0.354; 1.686			
Trunk FM (kg)	SIT	−0.8 ± 0.2	0.001	−1.145; −0.355	1.675	0.209	0.122
	HIIT	−0.6 ± 0.2	0.005	−0.917; −0.183			
	CG	−0.3 ± 0.2	0.203	−0.645; 0.145			
BP (mmHg)	SIT	03.1 ± 5.7	0.597	−8.809; 14.967	2.406	0.114	0.179
	HIIT	−13.4 ± 5.0	0.013	−23.773; −3.101			
	CG	−4.2 ± 5.7	0.462	−15.991; 7.509			
SBP (mmHg)	SIT	6.3 ± 6.7	0.357	−7.563; 20.114	2.202	0.134	0.167
	HIIT	−12.4 ± 5.8	0.044	−24.405; −0.341			
	CG	−4.2 ± 6.6	0.532	−17.862; 9.493			
DBP (mmHg)	SIT	1.5 ± 6.3	0.817	−11.653; 14.614	1.781	0.192	0.139
	HIIT	−14.0 ± 5.5	0.019	−25.388; −2.55			
	CG	−4.3 ± 6.3	0.502	−17.250; 8.711			
PR (bpm)	SIT	−4.7 ± 3.5	0.196	−11.995; 2.610	2.280	0.126	0.172
	HIIT	−10.5 ± 3.1**	0.002	−16.837; −4.139			
	CG	−0.8 ± 3.5	0.815	−8.04; 6.394			
Chol (mg/dl)	SIT	−1.3 ± 3.9	0.743	−10.431; 7.756	0.038	0.850	0.005
	HIIT	−2.4 ± 3.6	0.526	−10.683; 5.911			
LDL (mg/dl)	SIT	4.5 ± 5.3	0.422	−7.77; 16.778	6.739	0.032	0.457
	HIIT	−14.3 ± 4.9	0.019**	−25.452; −3.055			
HDL (mg/dl)	SIT	−0.4 ± 2.1	0.844	−5.343; 4.475	4.497	0.067	0.360
	HIIT	5.7 ± 1.9	0.019	1.216; 10.174			
Trig (mg/dl)	SIT	−30.7 ± 31.9	0.365	−104.355; 42.982	2.094	0.186	0.207
	HIIT	32.1 ± 29.1	0.303	−35.143; 99.286			
LipidNonHDL (mg/dl)	SIT	−1.5 ± 3.5	0.683	−9.617; 6.632	1.902	0.205	0.192
	HIIT	−8.1 ± 3.2	0.036	−15.502; −0.677			
Chol/HDL (mg/dl)	SIT	0.0 ± 0.1	0.919	−0.168; 0.153	7.181	0.028	0.473
	HIIT	−0.3 ± 0.1	0.003	−0.407; −0.114			
VO^2^max (ml/kg/min)	SIT	1.0 ± 0.6	0.123	−0.298; 2.330	3.319	0.056	0.240
	HIIT	2.2 ± 0.6	0.002*	0.916; 3.544			
	CG	−0.0 ± 0.6	0.990	−1.247; 1.232			

BMI, body mass index; BP: blood pressure; Chol, cholesterol; DBP, diastolic blood pressure; FM, fat mass; HDL, high density lipoprotein; LDL, low density lipoprotein; PR, pulse rate; SBP, systolic blood pressure; Trig, triglycerides.#Tendency towards significance from corresponding CG value at the *p* < 0.07 level. *Significantly different from corresponding CG value at the *p* < 0.05 level. ** Significantly different from corresponding CG value at the *p* < 0.01 level.

The univariate analysis of the mean differences showed a significant difference between HIIT and CG in the pre-post test change in VO^2^max (*p* = 0.047). HIIT not only showed a significant improvement while the CG and SIT showed no change, but this change was significantly different to that shown by the CG as well. The same was observed in relation to BP, where HIIT showed a significant change in BP, while SIT and CG showed no change, with this change being significantly different from that shown by SIT (*p* = 0.04). Similar results were observed between SIT and HIIT in LDL (*p* = 0.35) in favour of HIIT (*p* < 0.05).

The PACES questionnaire score reported an improvement in enjoyment of practicing physical activity with respect to the enjoyment of previous physical education sessions, in the total sample of 0.4 points (*p* = 0.026). Both groups showed improvement in enjoyment (SIT: diff. Pre-post = 0.41; *p* = 0.11; HIIT: diff. Pre-post = 0.38; *p* = 0.12), although it was not significant. Likewise, no significant difference was reported in the pre-posttest change between SIT and HIIT (t = 0.09; *p* = 0.93). In both cases, above average satisfaction was reported in both experimental groups (Table 4).

**Table 4:** Differences between groups in the change pre-post intervention and group*time interaction for PACES questionnaire adjusted by sex.

Outcome	Group	Pre-test (M ± ED)	Post-test (M ± ED)	Adjusted by sex			Group * time interaction		
				Difference post-pre (M ± ED)	*p*	95% CI (Mpost-Mpre)	*F*	Sig	ES
PACES	SIT	2.7 ± 0.1	3.1 ± 0.2	0.3 ± 0.2	0.191	−0.179; 0.818	0.166	0.690	0.012
	HIIT	2.9 ± 0.1	3.3 ± 0.2	0.5 ± 0.2	0.057	−0.016; 0.921			
	Total	2.8 ± 0.1	3.2 ± 0.1	0.4 ± 0.2	0.026	0.053; 0.719			

PACES, Physical Activity Enjoyment Scale; EG, experimental group.

Finally, the generalizability analysis (Table 5) shows a generalizability coefficient (GC) of 0.97 for the first design. This result shows a very high test–retest reliability. The percentage of variance was high in all tests.

**Table 5:** Absolute generalisability coefficient, relative generalisability coefficient, absolute standard deviation, and relative standard deviation in each of the designs.

Absolute generalisability coefficient	Relative generalisability coefficient	Absolute standard deviation	Relative standard deviation
0.955	0.967	0.315	0.267

Sum of squares	DF	Mean squares	%	Standard error
325.463	285	1.142	31.076	0.095

## 4 Discussion

The main aim of this study was to evaluate the effect of SIT and HIIT carried out during the cool-down period of the physical education classes during school hours on body composition, BP variables and PR, and cardiorespiratory fitness of adolescents who are overweight and obese, and to compare the differences in enjoyment in response to SIT vs. HIIT. A significant finding of this research was that both intervention groups showed a decrease in total FM and trunk FM. In this sense, a systematic review with meta-analysis in adults also found no differences in the effect of different HIIT protocols in overweight and obese adults (12). However, few studies had analysed this issue in overweight and obese adolescents. Previous studies have found that although HIIT is generally an effective training method for reducing fat mass, in many cases in this type of population, the intensity with which HIIT is performed is not sufficient to induce these changes (12). In line with this previous finding, in the present research, HR was monitored during all sessions to monitor the intensity at which the participants performed the exercises, which could have allowed them to work at the prescribed intensities, maximising the chances of obtaining changes in body composition, in line with previous literature (9, 12, 34).

Few studies have pointed to the ability of HIIT to induce changes in lean mass (34), even when working with self-loading, as was the case in the present research (35). However, these previous studies had not analysed which type of HIIT protocol would be most effective. The novelty of the present study is that the effects on lean mass were found with HIIT but not with SIT. The observed advantages of the HIIT protocol in lean mass may be attributed to the dose–response relationship between muscle hypertrophy and training volume. Higher training volumes can lead to greater gains in muscle mass, with muscle failure promoted through the use of a low-load such as self-loads (36). Based on these findings, HIIT may be a promising approach for optimizing healthy changes with respect to lean mass in overweight and obese adolescents.

A surprising finding of the present research was that males showed a greater improvement than females for fat percentage and visceral fat. Few previous studies have examined sex differences in the effects of HIIT (12, 37). However, to our knowledge, this is the first study to show that the effects on body composition after performing HIIT-based training depends on sex, in adolescents around the age at peak height velocity (APHV). Previous studies have shown that hormonal changes around APHV, related to the maturational process, have significant effects on body composition, with an increase in muscle mass and a decrease in fat mass found in males; and an increase in fat mass in females (38, 39). Thus, this aspect could be modulating the effects of HIIT on body composition, so results of current research must be interpreted with some reservations due to maturation has not been controlled, among other polluting factors. Therefore, considering the results of the present study, HIIT could be an effective training programme for achieving fat mass reduction in overweight and obese adolescents around the APHV, even for females.

Another main finding of this research was about the effect of SIT and HIIT on BP variables and PR. Previous reviews that analysed the effects of HIIT on BP variables and PR in children and adolescents (18), and in overweight and obese children and adolescents (9), found contradictory results. The authors concluded that this could be due to differences between the prescribed HIIT protocol (9, 18). In the present research, it was observed that HIIT significantly improved in BP variables and PR, whereas no changes were observed when the adolescents performed SIT. Only one previous study analysed HIIT vs. SIT in obese pre-adolescent boys. No adaptations were found in the majority of cardiometabolic physiological factors (19). However, this study has some limitations, such as the fact that it compared two training programmes with different training volumes, or training with a cyclometer, and this type of HIIT has been shown to be less effective than that based on running (12), which was the method chosen in the present study.

Another finding of this research was about the effect of SIT and HIIT on cardiorespiratory fitness in adolescents. Several systematic reviews with meta-analyses on the effects of HIIT in children and adolescents (40), and overweight and obese children and adolescents (9) found that most of the studies showed an improvement in aerobic fitness with HIIT, although there were also some studies that found no adaptations, and the divergence between the results found may be due to differences in the intervention programmes applied. To our knowledge, no previous studies have compared the effects of different HIIT protocols on cardiorespiratory fitness in overweight and obese adolescents. The present study showed that a HIIT programme led to a significance improvement in VO^2^max, whereas SIT appeared to have no effect. The absence of changes in SIT could be attributed to the intervals shorter than 30″ being energetically covered with the muscle cells’ oxygen stored in myoglobin, which helps sustain the exercise intensity without relying heavily on external sources of oxygen (41). In this sense, previous studies have pointed out that with intervals lasting less than 2′ it is difficult to reach VO^2^max, due to the kinetics of the VO2 response (42), which would minimise the adaptations on this parameter (43).

An important influence on physical activity adherence is the level of perceived exercise enjoyment (18). A noteworthy result of the present research is that an improvement in enjoyment during activity was found in participants who performed both SIT and HIIT. These results are consistent with those found in previous studies that implemented a HIIT programme with overweight adolescents (44). However, in spite of these promising results, insufficient literature confounds our ability to draw conclusions. Thus, there is a broad scope for research to examine if HIIT desirability in other adolescents who are overweight and obese depends on the training protocol.

Based on the above, the initial hypothesis can be partially accepted. On the one hand, most of the body composition variables showed improvements in both the SIT and HIIT groups, compared to the control group. HIIT protocol also showed a significant improvement of BP variables, PR and cardiorespiratory fitness. On the other hand, the SIT protocol had no significant influence on BP variables, PR and cardiorespiratory fitness, which makes it necessary to reject the hypothesis concerning this training protocol. Furthermore, there was no clear hypothesis as to whether the SIT or HIIT protocol would be more effective in generating adaptations on body composition, BP variables, PR and cardiorespiratory fitness and, considering the results of the present investigation, it appears that HIIT may be more effective in this area and in both sexes.

## 5 Limitations, strengths and practical implications

This research is not without limitations. The first limitation is the size of the sample and the absence of an assessment of maturational status, although this factor could be conditioning body composition, cardiorespiratory fitness and adaptations to exercise (39). The second limitation is the way of classifying the population as overweight/obese. This classification was made according to the BMI criterion, as this is the most commonly used parameter for this issue (24, 45). Another alternative could have been to use bioimpedance values relative to fat mass, but previous research has pointed out that the bioimpedance instrument used (46, 47) and the software and formulas used in them (48) could have a significant influence on the reported result. Therefore, in the present investigation, bioimpedance was discarded as an instrument to classify overweight/obesity. However, future research could contrast the results of this research using other methods to classify overweight/obesity. Finally, the third limitation of this research was that the effect of the covariate sex on the changes obtained with the HIIT and SIT programme was analysed in the present study, but due to the size of the sample, it was not possible to divide it into groups according to sex. Therefore, this is an issue to be addressed in future research.

Despite these limitations, strengths can be marked as this is the first study to analyse the effects of SIT and HIIT protocols in overweight and obese adolescents. Nevertheless, a strength of the present study is the large number of objective variables related to health utilized, for a comprehensive point of view.

Regarding the practical implications of the present research, the implementation of a HIIT programme of 12 min per session, carried out during the cool-down period of physical education classes, two days a week, could be a good resource to achieve an improvement in body composition, cardiorespiratory fitness, BP variables and PR in overweight and obese adolescents. This should be taken into account by educational institutions and physical education teachers, as it could improve the health of this population. However, a SIT protocol may not be as effective in this population.

## 6 Conclusion

The present study found that a HIIT protocol performed during the cool-down period of the physical education classes generated adaptations such as improvement in body composition, BP variables, PR, and cardiorespiratory fitness, in overweight and obese adolescents. In contrast, the group of overweight and obese adolescents who performed SIT showed limited benefits, with changes in fat mass only. This is the first study to analyse the effects of SIT and HIIT protocols in overweight and obese adolescents. However, given the limitations of the present study and the small sample size, it is necessary to replicate this study in the future to be able to contrast and generalise the results found.

## Data availability statement

The raw data supporting the conclusions of this article will be made available by the authors, without undue reservation.

## Ethics statement

The studies involving humans were approved by Institutional Ethics Committee of the San Antonio de Murcia Catholic University (code: CE061914). The studies were conducted in accordance with the local legislation and institutional requirements. Written informed consent for participation in this study was provided by the participants' legal guardians/next of kin.

## Author contributions

NG-G: Conceptualization, Data curation, Formal analysis, Funding acquisition, Investigation, Methodology, Project administration, Resources, Software, Supervision, Validation, Visualization, Writing – original draft, Writing – review & editing. AS-M: Formal analysis, Investigation, Resources, Visualization, Writing – original draft, Writing – review & editing. TA-L: Data curation, Investigation, Writing – original draft, Writing – review & editing. LA-C: Data curation, Investigation, Writing – original draft, Writing – review & editing. AM-O: Investigation, Writing – original draft, Writing – review & editing. RV-C: Investigation, Resources, Validation, Visualization, Writing – original draft, Writing – review & editing.
